# Investigation of Information Overload in Electronic Health Records: Protocol for Usability Study

**DOI:** 10.2196/66127

**Published:** 2025-02-11

**Authors:** Saif Khairat, Jennifer Morelli, Marcella H Boynton, Thomas Bice, Jeffrey A Gold, Shannon S Carson

**Affiliations:** 1 School of Nursing University of North Carolina at Chapel Hill Chapel Hill, NC United States; 2 School of Medicine University of North Carolina at Chapel Hill Chapel Hill, NC United States; 3 School of Medicine Oregon Health & Science University Portland, OR United States

**Keywords:** electronic health records, information overload, eye-tracking, EHR usability, EHR interface

## Abstract

**Background:**

Electronic health records (EHRs) have been associated with information overload, causing providers to miss critical information, make errors, and delay care. Information overload can be especially prevalent in medical intensive care units (ICUs) where patients are often critically ill and their charts contain large amounts of data points such as vitals, test and laboratory results, medications, and notes.

**Objective:**

We propose to study the relationship between information overload and EHR use among medical ICU providers in 4 major United States medical centers. In this study, we examined 2 prominent EHR systems in the United States to generate reproducible and generalizable findings.

**Methods:**

Our study collected physiological and objective data through the use of a screen-mounted eye-tracker. We aim to characterize information overload in the EHR by examining ICU providers’ decision-making and EHR usability. We also surveyed providers on their institution’s EHR to better understand how they rate the system’s task load and usability using the NASA (National Aeronautics and Space Administration) Task Load Index and Computer System Usability Questionnaire. Primary outcomes include the number of eye fixations during each case, the number of correct decisions, the time to complete each case, and number of screens visited. Secondary outcomes include case complexity performance, frequency of mouse clicks, and EHR task load and usability using provided surveys.

**Results:**

This EHR usability study was funded in 2021. The study was initiated in 2022 with a completion date of 2025. Data collection for this study was completed in December 2023 and data analysis is ongoing with a total of 81 provider sessions recorded.

**Conclusions:**

Our study aims to characterize information overload in the EHR among medical ICU providers. By conducting a multisite, cross-sectional usability assessment of information overload in 2 leading EHRs, we hope to reveal mechanisms that explain information overload. The insights gained from this study may lead to potential improvements in EHR usability and interface design, which could improve health care delivery and patient safety.

**International Registered Report Identifier (IRRID):**

DERR1-10.2196/66127

## Introduction

Electronic health records (EHRs) can be a source of information overload for providers; however, the mechanisms that explain overload and their link to patient safety are not understood. Information overload in the EHR is associated with missing critical information that affects the decision-making process [1-3]. Studies have relied on secondary data analysis or subjective measures to assess the effect of information overload on decision-making processes; however, the use of physiological and objective data to study information overload has shown great potential in other domains [4]. We propose using eye-tracking approaches coupled with objective patient safety measures to investigate current EHR design flaws [5-9]. These methods reveal mechanisms that explain overload, such as fatigue and degradation in performance. Furthermore, poor EHR interface design contributes to inefficiencies [10], frustration [11], and medication errors [12].

Although advantageous over paper-based documentation, EHR use is associated with new physician-related challenges that may increase medical errors [13]. EHR interface design can lead 50% of providers to make an error when ordering medication tapering [12]. In addition, too much information contributes to patient safety risks, such as 30% of providers missing test results in the EHR, leading to care delays [2,14]. Information overload increases cognitive load and error rates among physicians during clinical simulations. Common issues contributing to information overload included an overabundance of clinically irrelevant information, poor data presentation, and excessive alert notifications [15]. However, we have limited knowledge of the relationship between information overload and EHR usability.

Our study uses objective eye-tracking data along with self-reported task load and usability surveys to better understand the challenges Intensive care unit (ICU) providers face in using EHRs to provide patient care. By measuring providers’ performance, information seeking load, and information processing load, we aim to characterize information overload in the EHR by examining decision-making and usability outcomes.

## Methods

We conducted a multisite, cross-sectional usability assessment of information overload in 2 leading EHRs among medical ICU providers in 4 US medical centers. Between the 4 sites, 2 leading EHR systems are used. The ICUs at the medical centers vary in terms of the catchment area; however, all 4 medical centers in the study are considered level I trauma centers, each with level 3 ICU support. The number of ICU beds between the 4 medical centers ranges from approximately 74 to 200 ICU beds each.

Providers will be recruited by the individual study site teams at each medical system using emails, flyers, and word of mouth. Interested providers will be asked to complete an initial screening survey before scheduling a session to ensure that they are an ICU provider or, if they are a resident, that they have completed at least one ICU rotation.

The sessions will occur in a simulation laboratory or private space that mimics the ergonomics of natural inpatient settings. Our team used the standard computer screen in each practice setting, with ICU-like ergonomic placement, ambient lighting, and seating. To ensure standardization among the cases, the lead investigators at each site met via Zoom (Zoom Video Communications) to determine a rubric for patient records to be included in the study. The study team created 4 ICU patient cases for inclusion, based on reviews of our 2 domain experts. Next, the domain experts created a set of universal experimental questions and tasks for providers to complete for each patient.

Before each session, the usability specialist explained the study and consent forms to providers, assuring them that the goal is to assess EHR usability rather than clinical knowledge. Participants are informed that their participation is voluntary and that they can decline to participate at any time. After the informed consent has been obtained, each provider will be asked to complete 3 pages of paper surveys.

We then ask providers to complete a basic calibration exercise while looking at the monitor which allows the eye-tracking software to be calibrated to each provider’s eye shape and eye size. We record pupil diameter and fixation points on the screen continuously during the study session. All sessions used the same screen-based eye tracker.

**Figure 1:**
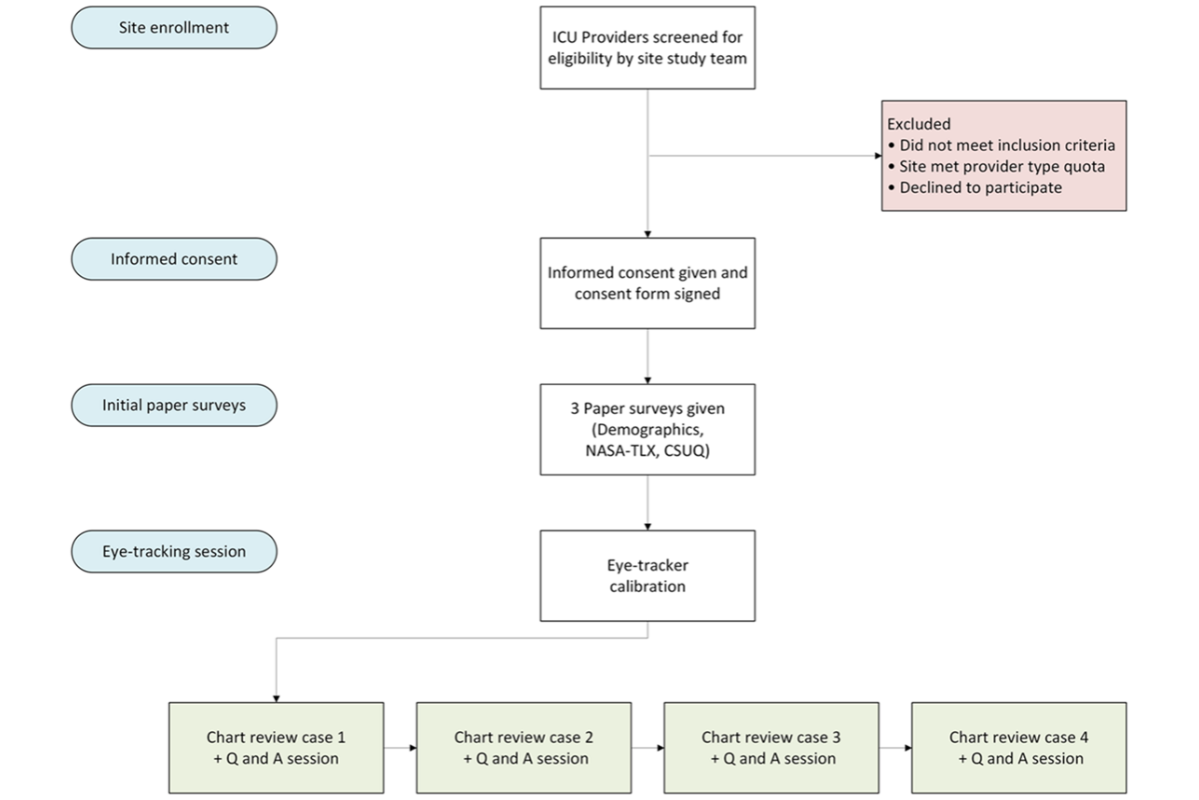
Process map illustrating the various study steps from recruitment to completion of the usability session. CSUQ: Computer System Usability Questionnaire; ICU: intensive care unit; NASA-TLX: National Aeronautics and Space Administration–Task Load Index; Q and A: question and answer.

Each provider was then logged in to their institution’s EHR environment, where 4 simulated ICU patient records are presented in random order to eliminate selection bias. Providers reviewed 1 patient case at a time and were encouraged to review the patient chart as if they were prerounding on this patient, using their typical workflow. The provider then indicated to the research assistant (RA) when they completed their chart review and were ready to begin a series of question-and-answer activities about that specific patient. The RA asked the provider a series of questions in which the provider responded verbally, and the RA recorded the answers using both audio and written recordings. Providers were allowed to navigate through the EHR to answer these questions. After completion of question-and-answer activities, the provider then reviewed the next patient record. A process map detailing the study procedure from enrollment to usability session can be seen in Figure 1.

### Patient Cases

A team of critical care physicians created 4 patient cases. Each patient was representative of a patient that could be hospitalized in an ICU setting. Of the 4 patients, 2 were considered basic or “standard” ICU patients while 2 were considered to be complex patients. The 2 basic patients were less critically ill, not currently ventilated, on fewer medications, and had fewer abnormal laboratory values. The 2 complex patients were critically ill with many abnormal laboratory values, on the ventilator with poor oxygenation, and on complicated medication regimens.

The nurse informatician on the study team worked with the primary investigator and each site’s study team to build the patient cases in their institution’s EHR test environment. The same 4 cases were used at all study sites, and the nurse informatician monitored case builds to ensure consistency and accuracy across study sites.

### Case Questions

Providers were asked 5 questions for each patient following their preliminary chart review. These questions were created by a critical care physician (TB) and reviewed by a critical care physician team before data collection. Questions were written to ensure the answer is present within the EHR and that providers at different levels of experience can realistically locate the answer. While some questions were considered more basic and only needed 1 EHR screen or data point to answer, other questions were more complex and required synthesizing multiple data points or locating information not frequently accessed. This approach allows us to analyze the time it takes to answer these questions and how many EHR screens and mouse clicks each provider uses before answering a question. Questions were scored as either correct, partially correct, or incorrect by the team nurse informatician. Alternate answers will be reviewed by a critical care physician (SC) for scoring.

### Sample

Our initial goal was to recruit 80 ICU providers, 20 at each site, with the following distribution: 15 physicians and 5 advance practice providers (APPs). The physician group will be divided into 3 subgroups: 5 attending physicians, 5 fellow physicians, 5 resident physicians, and 5 APPs including nurse practitioners and physician assistants. Our actual recruitment was 81 ICU providers, comprised of 53 physicians and 28 APPs. Each study team’s local RAs circulated departmental emails and flyers at each site. They provided interested individuals with a link to an online calendar showing available time slots to facilitate appointment scheduling. Once an appointment was scheduled, the RA emailed each participant their appointment time and location with a map, as well as a contact number for same-day inquiries or cancellations. Inclusion criteria: ICU physicians and APPs on active full-time ICU service OR Residents who have completed at least 1 ICU rotation, use an institutional EHR, and speak English.

### Statistical Power

The design effect arising from the clustering within-person inherent to the study’s design is estimated using the following formula: D_eff_=1+(m-1), where m=average cluster size (4 cases) and *P*=intraclass correlation coefficient (ICC). Assuming an ICC of .6 (a common ICC for similar intensive repeated measures designs), the application of this equation results in D_eff_ of 1.09, for an effective sample size of 294 (320/2.8). In a multivariable linear regression context assuming N=114, 5 person-level effects, 3 case level effects, a 2-tailed critical *P*=.05, and 80% power, the minimum detectable effect size is represented by a Cohen f^2^ of 0.10, which is considered a small effect. In a multiple logistic regression with 2 tailed critical *P*=.05 and 80% power, the minimum detectable odds ratio is 3.23, which is considered a medium-sized effect.

### Materials and Software

To measure the extent and effect of EHR information overload, we used several tools during the 1-hour sessions.

Providers were asked to complete 3 paper surveys before the eye-tracking session. The first survey asked basic demographic questions, including age, gender, years since graduation (medical school or APP schooling), years of experience with their institution’s EHR, and the estimated number of hours they use their institution’s EHR each week. We also asked the providers if they are on service that day (pre- or post-usability session) and to rate their level of sleepiness and stress on a Likert scale. The second paper survey used was the NASA (National Aeronautics and Space Administration) Task Load Index (NASA-TLX) which asked providers to rate the task load of their institution’s EHR, including how mentally and physically demanding it is to use and their level of satisfaction and confidence with using the EHR [16]. The third and final paper survey was the Computer System Usability Questionnaire (CSUQ) which asked providers to rate their satisfaction with their institution’s EHR system including questions about usability, ease of use, the system interface, and overall functionality [17].

Our study used a noninvasive screen-based eye tracker to provide further insights into a provider’s cognitive processing during the sessions. The Tobii Pro Fusion is an advanced eye-tracking system with 2 eye-tracking cameras taking up to 250 images per second to ensure accuracy. The Pro Fusion mounts onto the bottom of any monitor, allowing us to record eye-tracking data seamlessly. The Tobii eye-tracker was used with Tobii Pro Lab software to create a seamless recording that includes eye-tracking data and screen recording. Used by renowned researchers in medicine and psychology [18,19], the Tobii eye-tracker and software will provide us with fixation points, gaze areas, and eye movement type. These measures will provide us with insight regarding providers’ information processing behaviors, in particular, the relationship between fixation points (a measure of concentration) and the outcome variable (decision-making accuracy). We have expertise in analyzing large and complex data sets from Tobii, considered the most accurate eye-tracking device manufacturer in the world.

#### Outcomes

Primary outcomes are cognitive overload (ie, fixation points), usability (clicks and completion time), and performance score. Secondary outcomes are the provider reported EHR workload and usability using the NASA-TLX and computer system usability questionnaire surveys.

To characterize information overload, we will explore under what conditions providers experience EHR information overload. We will accomplish this by determining which patient cases create the highest level of information overload, indicated by the least number of eye fixations, meaning a loss of concentration. We also want to understand the consequences of a lack of concentration as it relates to decision accuracy and time. Understanding the underlying factors and the consequences of information overload will fill a significant gap in the literature.

#### Analytical Plan

Each participant will receive a single score for each question determined by the domain expert (0: incorrect, 0.5: partial, and 1: correct). Correct decisions are the aggregate of the correct answers, and errors are the aggregate of the incorrect answers. For each participant, we will compute a score for each patient case and a total score for all the patient cases. Case scores will depend on the number of questions asked, such that if a case has 5 questions to be answered, the total score of this case will be equivalent to the highest possible points (5).

Our past work will inform ways to compute case scores [10,12]. Responses and scores will be assigned by 2 domain experts (the site-Principal Investigator and a senior ICU provider).

Eye-tracking data gives the frequency and duration of fixations for each participant. We will calculate participants’ fixation points based on (1) total fixations for the study, (2) total fixations per patient case, and (3) total fixations per EHR screen visited.

We will compute descriptive statistics for patient and case characteristics, employing chi-square tests, *t* tests, and ANOVAs to examine between-group differences, where appropriate. We will examine differences between provider types (physicians and APPs), as well as between sites. Given the nested nature of the data (ie, repeated case assessments nested within providers), we will use multilevel modeling as the primary analytic approach. Each patient case will be coded based on the days of ICU stay and the presence or absence of key characteristics such as vent settings and intake and output, to determine the level of complexity. Case variables will be entered into the model as case-level (Level 1) predictors. Models will also account for person-level (Level 2) factors such as participant gender, age, clinical role, site, and years of EHR experience. We will use SAS (version 9.4; IBM Corp) using PROC MIXED for continuous outcomes and SAS PROC GLIMMIX for binary and count outcomes.

### Ethical Considerations

Recruitment and site testing occurred sequentially, such that the study was implemented at one site at a time, allowing the study team to be present on site to add organization to the data collection process. Each participant was required to read and sign a consent form specific to their medical center, witnessed by a representative of that medical center. Participants were allowed to opt out of the study at any time. We used one screen-based eye-tracker device for data collection at each site. We recruited through flyers and departmental email communications at each site. Participants were compensated with a US $100 gift card for their participation. All data collected were deidentified and study participants and their data were assigned a unique identification number. Study data entry and management systems are secured and password protected to ensure participant privacy and confidentiality. The University of North Carolina at Chapel Hill’s institutional review board (IRB) approved this study (IRB #20-3384).

## Results

This EHR usability study was funded in 2021. The study was initiated in 2022 with a completion date of 2025. Data collection was completed in December 2023 with a total of 81 provider sessions recorded. The primary analysis is ongoing and expected to be published in early 2025.

## Discussion

### Study Significance and Strengths

This study aims to characterize information overload in the EHR and uncover possible improvements to EHR interfaces and user skills. We will deploy a mixed methods approach to better study providers’ reactions to information overload in the EHR. We will use usability evaluation metrics, eye-tracking, and surveys to assess the aforementioned relationship. The use of physiologic data, namely eye-tracking, will produce new knowledge about providers’ cognitive performance during EHR interaction. Eye-tracking will allow us to quantify fixation points, a measurement of cognitive overload, as providers interact with basic and complex ICU patient cases. In this study, we examine the 2 most prominent EHR systems in the US, which will generate reproducible and generalizable findings that can be applied to clinical settings using an EHR system. Another strength of this study is the inclusion of different professional roles, including residents, APPs, fellows, and attending. This diverse sample will enable subgroup analysis to test if information overload has similar effects on different professional roles.

Examining information overload within EHRs will demonstrate the critical impact of EHR usability on clinical decision-making, especially in high-pressure environments like ICUs. By measuring and assessing information overload, we can identify design opportunities in EHRs that may improve providers’ ability to access and process vital information. This knowledge enables health systems to enhance patient safety and the quality of care by tailoring training and support systems that address specific challenges faced by providers. Future research needs to involve longitudinal studies to evaluate the long-term effects of information overload on clinical performance and patient outcomes. Incorporating qualitative methods, such as interviews with providers, will complement our quantitative findings to create a comprehensive understanding of EHR interactions. Potential interventions may include the implementation of information visualization within EHRs to facilitate information processing, implementing tailored training programs for providers, and developing integrated decision support tools. These research goals aim to enhance EHR usability, reduce the cognitive burden on health care professionals, and promote a safer and more efficient health care delivery system.

### Limitations

Although we proposed adequate recruitment numbers, we understood that recruiting the exact number of participants in each professional role may be challenging. Alternatively, we expanded recruitment to include medical ICUs in other affiliated hospitals within the same health care system. All study sites include multiple hospitals to enable expanding recruitment within the same system and under the same IRB. Potential bias may occur from recruiting participants who are technology enthusiasts. We attempted to mitigate this bias by predefining a quota for every professional role to ensure we have representation from junior and senior providers. This mix of roles was done to include persons with varying degrees of technological astuteness.

Variations in EHR design and performance may affect the study findings. We account for this limitation by including the 2 most prominent EHR systems and 4 different medical centers, leading to more generalizable findings. The study protocol was not published earlier to keep the study design and the components of the patient cases undisclosed to avoid introducing bias to potential participants.

### Future Directions

We will use our findings from this study to guide future research on information overload in the EHR. Observations of the current opinions and functionality of each of the 2 institutional EHRs will be used to prepare for a randomized controlled trial using the same 4 US medical centers. This randomized controlled trial will compare the current institutional EHR’s interface with a visualization dashboard, examining the differences in provider efficiency and fatigue.

### Conclusions

Our study aims to characterize information overload in the EHR by examining decision-making and usability outcomes among medical ICU providers. By conducting a multisite, cross-sectional usability assessment of information overload in leading EHRs, we hope to reveal mechanisms that explain overload, such as fatigue and degradation in performance. Through the use of eye-tracking approaches coupled with objective patient safety measures, we aim to investigate current EHR design flaws and their impact on decision-making processes. The insights gained from this study will contribute to a better understanding of the relationship between information overload, EHR usability, and patient safety, ultimately leading to potential improvements in EHR interface design and health care delivery.

## Abbreviations

APP: advance practice provider
CSUQ: Computer System Usability Questionnaire
EHR: electronic health record
ICC: intraclass correlation coefficient
ICU: intensive care unit
IRB: institutional review board
NASA-TLX: National Aeronautics and Space Administration–Task Load Index
RA: research assistant

## Data Availability

The data that support the findings of this study are not openly available due to reasons of sensitivity and are available from the corresponding author upon reasonable request. Data are located in controlled access data storage at the University of North Carolina at Chapel Hill.
